# Whey Protein Concentrate Renders MDA-MB-231 Cells Sensitive to Rapamycin by Altering Cellular Redox State and Activating GSK3β/mTOR Signaling

**DOI:** 10.1038/s41598-017-14159-5

**Published:** 2017-11-21

**Authors:** Shih-Hsuan Cheng, Yang-Ming Tseng, Szu-Hsien Wu, Shih-Meng Tsai, Li-Yu Tsai

**Affiliations:** 1 Department of Medical Laboratory Science and Biotechnology, College of Health Sciences, Kaohsiung Medical University, No. 100, Shih-Chuan 1st Rd., Kaohsiung, 80702 Taiwan; 2 Department of Pathology and Laboratory Medicine, Kaohsiung Veterans General Hospital, No. 386, Ta-chung 1 Rd., Kaohsiung, 81346 Taiwan; 3 Division of Plastic Surgery, Department of Surgery, Taipei Veterans General Hospital, No. 201, Sec. 2, Shipai Rd., Taipei, 11221 Taiwan; 4 Department of Surgery, School of Medicine, National Yang Ming University, No. 155, Sec. 2, Linong Street, Taipei, 11221 Taiwan; 5 Department of Environmental and Public Healthy, School of Medicine, College of Medicine, Kaohsiung Medical University, No. 100, Shih-Chuan 1st Rd., Kaohsiung, 80702 Taiwan

## Abstract

Whey protein concentrate (WPC) is an amino acid-rich supplement that has been shown to increase cellular antioxidant capacity. Mammalian target of rapamycin (mTOR) is a crucial regulator of signaling in mammalian cells, and serves as a therapeutic target for triple-negative breast cancer (TNBC). This study was designed to investigate the effect of combining WPC with rapamycin on MDA-MB-231 human breast cancer cells. These cells were found to be insensitive to rapamycin and exhibited higher glutathione (GSH) and reactive oxygen species levels than non-tumorigenic MCF-10A cells. However, for MDA-MB-231 cells, the half maximal inhibitory concentration of rapamycin was lower when this drug was administered in combination with WPC than when used alone. Furthermore, combining WPC with rapamycin depleted GSH levels and reduced Nrf2 nuclear accumulation. In addition, WPC activated GSK3β/mTOR signaling, and GSK3β appeared to be involved in the WPC-mediated Nrf2 reduction and mTOR activation. In conclusion, WPC induced rapamycin sensitivity in MDA-MB-231 cells by altering their redox state and activating GSK3β/mTOR signaling. These results not only suggest a novel therapeutic approach for breast cancer treatment, but also provide insight into the critical pathways affecting the resistance to mTOR inhibition observed in a subgroup of TNBC patients.

## Introduction

Breast cancer is the most common malignancy affecting women worldwide and its incidence has increased globally over recent decades. Triple-negative breast cancer (TNBC), defined by the absence of estrogen receptor, progesterone receptor, and human epidermal growth factor receptor expression, remains a therapeutically challenging disease with the worst prognosis of any breast cancer subtype^1^.

Mammalian target of rapamycin (mTOR), a downstream protein of the PI3K/Akt pathway, is activated by growth, changes in nutrient and energy levels, and hypoxia, and has also been implicated in cancer cell proliferation and survival^2^. mTOR complex 1 (mTORC1) responds to intracellular energy levels and nutrient availability, and once activated, phosphorylates and activates ribosomal p70 S6 kinase (p70S6K) and hyperphosphorylates eukaryotic translation initiation factor 4E (eIF4E)-binding proteins 1 (4E-BP1), influencing cell growth and survival. Recent evidence has indicated that glycogen synthase kinase 3 beta (GSK3β) positively regulates mTORC1 activity in MCF-7 breast cancer cells^3^. Moreover, GSK3β has been shown to play a permissive role in the amino acid-induced activation of mTORC1^4^.

Reactive oxygen species (ROS) and cellular oxidative stress are associated with cancer^5,6^, and compared to normal cells, antioxidant capacity is upregulated in malignant cells to adapt to higher ROS levels^5^. Previous studies have reported that nuclear factor (erythroid-derived 2)-like 2 (Nrf2) responds to increased ROS levels by enhancing the expression of genes involved in maintaining the cellular redox balance, including those encoding glutamate-cysteine ligase (GCL) and glutathione reductase (GR), which are associated with glutathione (GSH) production and regeneration^7^. Furthermore, deregulation of Nrf2, such as that leading to its increased nuclear accumulation, reduces apoptosis and promotes drug resistance^8^.

Since mTOR and Nrf2 signaling are known drivers of human oncogenesis^9,10^, agents targeting these pathways have shown promise as treatments for breast cancer^11,12^. However, despite the establishment of this treatment strategy and its encouraging results, a proportion of patients with TNBC harbor tumors that are resistant to mTOR inhibitors^13^. Thus, identifying markers of mTOR inhibitor sensitivity or the development of combination therapy is urgently needed to improve TNBC response to mTOR inhibition.

Whey protein concentrate (WPC) is prepared in a special manner to preserve native forms of the cysteine-rich proteins in whey (serum albumin, lactoferrin, and α-lactalbumin) and functions as a GSH precursor in cells. We have shown previously that WPC supplementation increases antioxidant activity in human peripheral blood mononuclear cells and rats treated with high doses of alcohol^14,15^. Besides, we have demonstrated that WPC supplementation selectively depletes tumor GSH levels in 7,12-dimethylbenz[a]anthracene (DMBA)-induced mammary tumors in rats^16^. In addition, prior studies have reported that WPC ingestion promotes activation of the mTOR pathway^17^, and it was demonstrated that this supplement exerts its antioxidant effects through an Nrf2-dependent mechanism in endothelial cells^18^. Therefore, in the present work, we aimed to determine whether WPC can influence the susceptibility to mTOR inhibitors using MDA-MB-231 TNBC cells, a cell line that has been reported to be resistant^19,20^. These results might provide insight into the critical pathways that are involved in resistance to mTOR inhibition in TNBC, and could identify biomarkers of for the responsiveness to such inhibitors.

## Results

### Determination of cellular redox status

**Figure 1:**
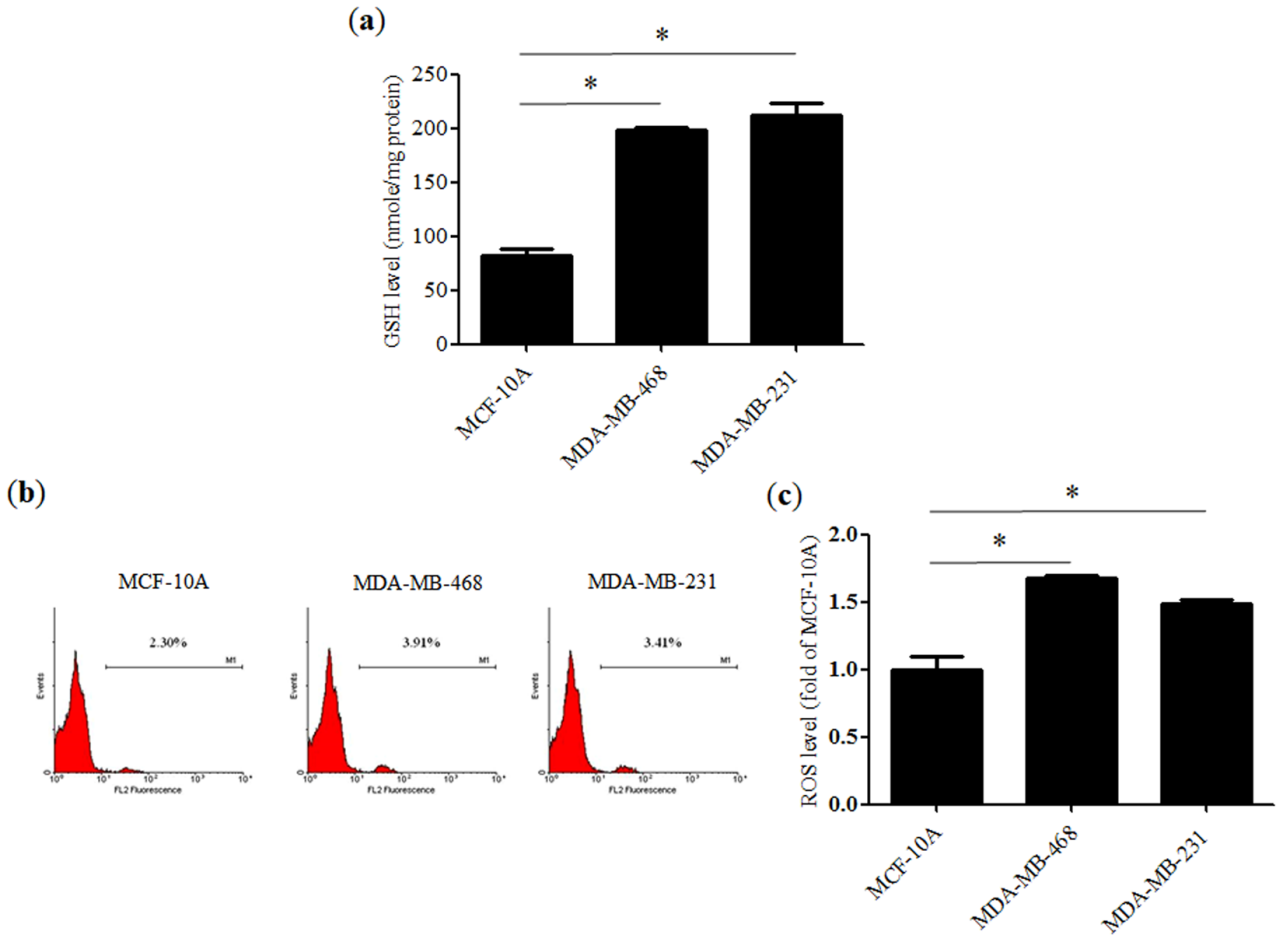
Redox status of various cell lines. (**a**) Glutathione (GSH) levels in non-tumorigenic MCF-10A cells and advanced breast cancer cell lines including MDA-MB-463 and MDA-MB-231 cells. (**b**) Representative flow cytometry histograms showing cellular reactive oxygen species (ROS) content and (**c**) a bar graph indicating fold-change relative to MCF-10A. Data represent the means ± SD from three experiments. *P < 0.05.

Increased GSH (Fig. 1a) and ROS (Fig. 1b,c) levels were noted in advanced breast cancer cell lines, including MDA-MB-231 and MDA-MB-468, compared to those in MCF-10A non-tumorigenic breast epithelial cells. Since GSH is a key cellular antioxidant, these results indicated that cancer cells have higher tolerance to ROS.

### WPC sensitizes MDA-MB-231 cells to rapamycin

**Figure 2:**
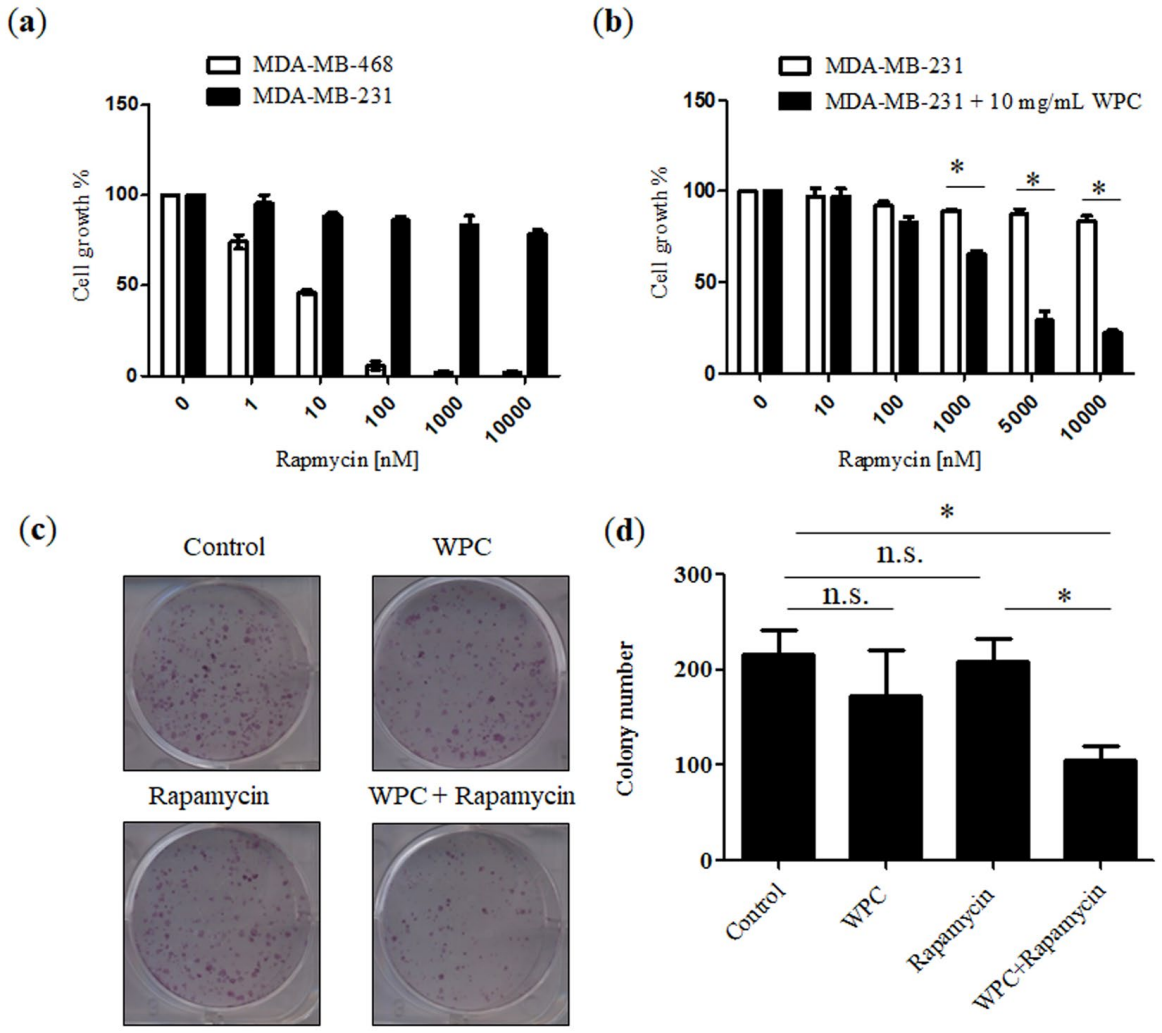
Whey protein concentrate (WPC) renders MDA-MB-231 cells sensitive to rapamycin and inhibits the colony formation of MDA-MB-231 cells. (**a**) MDA-MB-468 and MDA-MB-231 cell viability was determined by MTT assay after incubation with rapamycin at the indicated concentration for 72 hours. (**b**) Inhibition of MDA-MB-231 cell growth following 48 hours of exposure to 10 mg/mL WPC combined with 10, 100, 1,000, 5000, and 10,000 nM rapamycin. (**c**) Representative images demonstrating the effect of WPC, rapamycin, and their combination on colony formation in MDA-MB-231 cells. (**d**) Bar graph indicating the number of colonies formed. Data represent the means ± SD from three experiments. *P < 0.05; n.s.: not significant.

To examine the sensitivity of each cell line to rapamycin, cells were treated with this drug at different concentrations for 72 hours, and cell viability was measured by standard MTT assays. As shown in Fig. 2a, growth of MDA-MB-468 cells was significantly inhibited by rapamycin, whereas MDA-MB-231 cells were resistant to the growth inhibitory effects of this drug, with observed half maximal inhibitory concentration (IC_50_) values of 8.91 nM and >10 μM, respectively. Given these different responses to rapamycin, we chose the MDA-MB-231 line for further evaluation. In addition, cellular viability of MCF-10A, MDA-MB-468, and MDA-MB-231 cells was measured with WPC at concentrations of 1, 10, and 20 mg/mL. There were no significant differences (P > 0.05) in cell viability among the WPC-treated groups, despite increasing concentrations of WPC (Fig. S1a–c). Regarding viability of MDA-MB-231 cells, Fig. 2b shows the effect of 48-hour exposure to WPC (10 mg/mL) combined with different concentrations of rapamycin. Compared to treatment with rapamycin alone, the growth inhibitory effect of combined treatment resulted in a significant decrease in the IC_50_ of rapamycin (from >10 μM to 2.54 μM, respectively). *In vitro* colony formation assays were then performed to further test the effect of WPC combined with rapamycin on cell growth. Figure 2c and d demonstrate that combined treatment led to a significant decrease in the number of colonies compared to that in the control and rapamycin-only groups. However, cells treated with WPC or rapamycin alone did not significantly differ from the control in this respect.

### Effect of WPC, rapamycin, and their combination on cellular redox status

**Figure 3:**
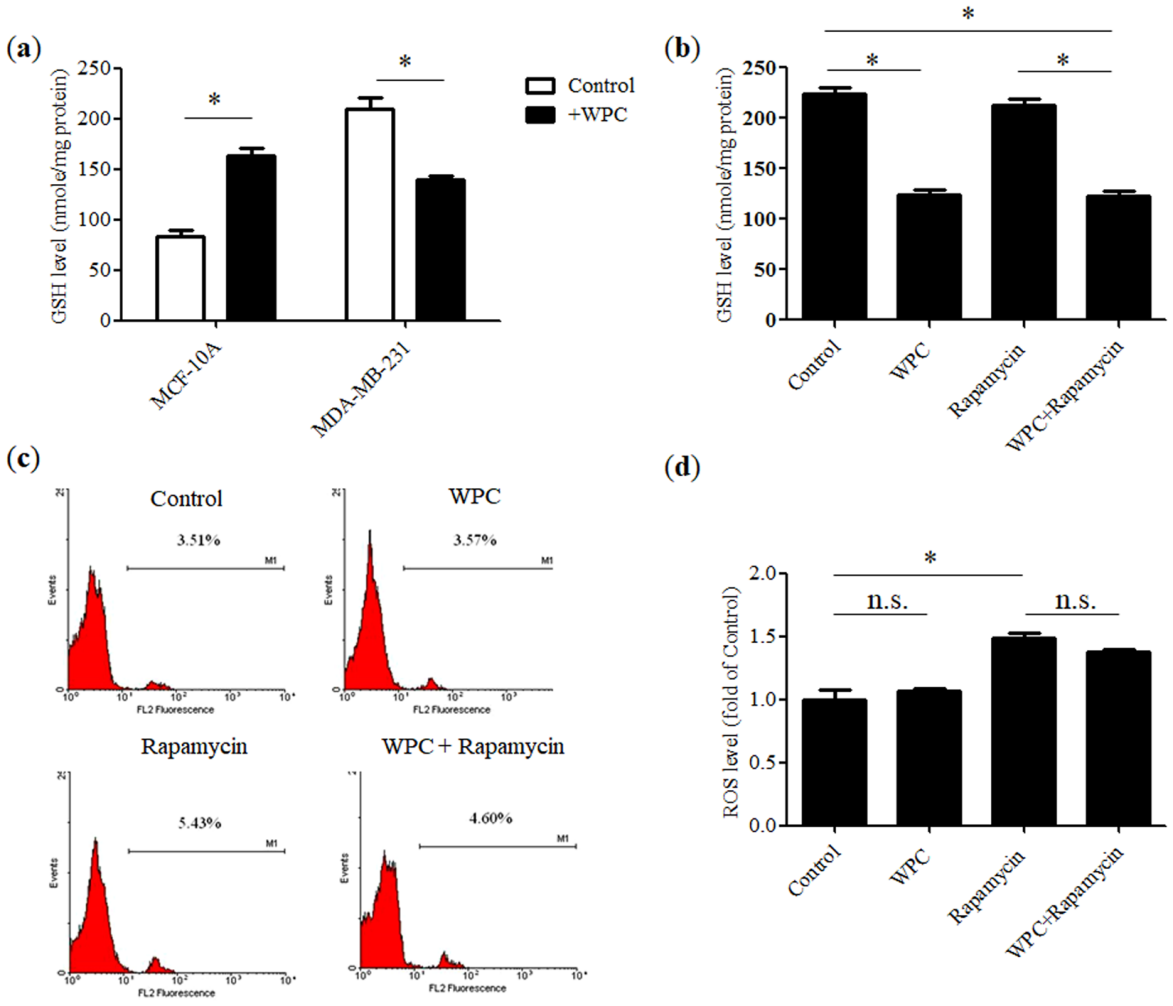
Effect of whey protein concentrate (WPC), rapamycin, and their combination on the redox status of breast cancer cells. (**a**) Glutathione (GSH) levels in non-tumorigenic MCF-10A cells and MDA-MB-231 cells after incubation with 10 mg/mL WPC for 48 hours. Effect of 48-hour treatment with 10 mg/mL WPC, 1 μM rapamycin, and their combination on (**b**) GSH levels and (**c**) reactive oxygen species (ROS) content in MDA-MB-231 cells, displayed as representative flow cytometry histograms and (**d**) a bar graph indicating fold-change relative to control levels. Data represent the means ± SD from three experiments. *P < 0.05; n.s.: not significant.

As shown in Fig. 3a, WPC (10 mg/mL) treatment selectively depleted GSH levels in MDA-MB-231 cells, but increased GSH levels in non-tumorigenic MCF-10A cells. Moreover, in MDA-MB-231 cells, rapamycin (1 μM) treatment did not alter GSH levels (Fig. 3b), but did significantly increase ROS concentrations (Fig. 3c,d) compared to those in the control. Furthermore, in comparison to those in the control, GSH levels were significantly lower following administration of WPC alone or in combination with rapamycin. However, WPC alone had no effect on ROS levels in MDA-MB-231 cells and did not affect the rapamycin-mediated elevation in ROS concentrations when administered as part of the combination treatment.

### Effect of WPC, rapamycin, and their combination on the expression of Nrf2, GCL, and GR in MDA-MB-231 cells

**Figure 4:**
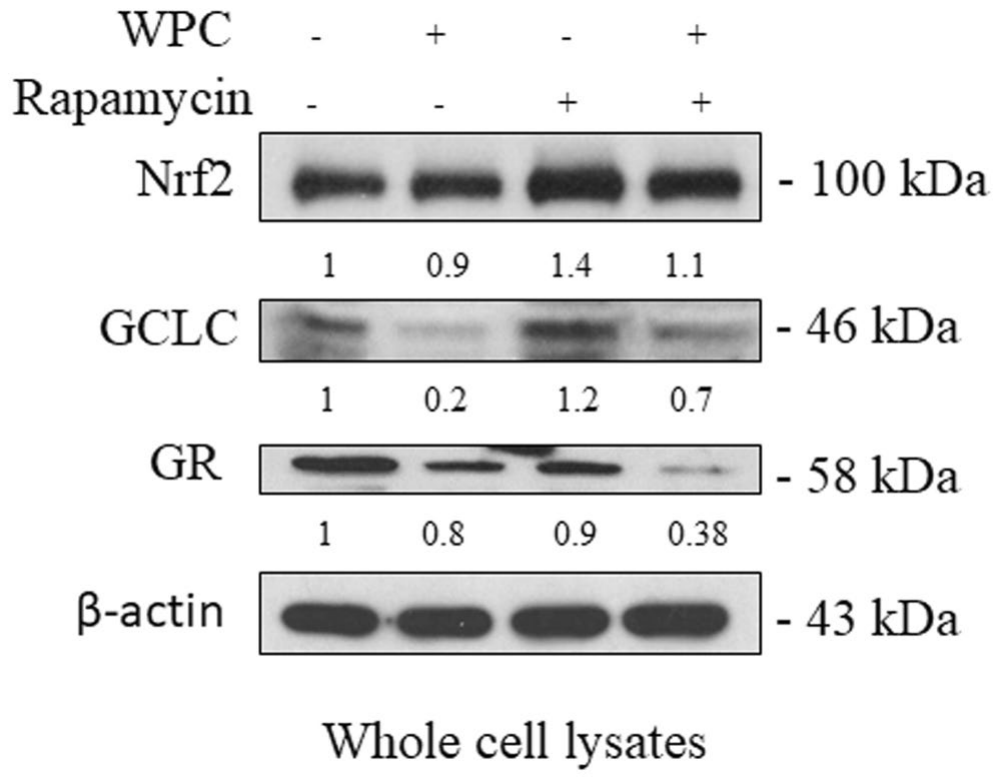
Effect of whey protein concentrate (WPC), rapamycin, and their combination on the expression of Nrf2, GCLC, and glutathione reductase (GR) proteins in MDA-MB-231 cells. Representative western blots showing the effect of 48 hours of exposure to 10 mg/mL WPC, 1 μM rapamycin, and their combination on whole cell Nrf2, GCLC, and GR protein levels. β-actin was used as a loading control. Data represent the means ± SD from three experiments.

Since the intracellular GSH redox state is regulated by the Nrf2-GCL-GR-GSH signaling pathway, whole cell lysates were subjected to immunoblotting for Nrf2, the GCL catalytic subunit (GCLC), and GR to clarify the effects of WPC. As shown in Fig. 4, there were no significant differences (P > 0.05) in whole-cell Nrf2 protein expression between groups with WPC treatment alone or combined treatment, when compared to that in the control group. However, treatment with rapamycin alone increased whole-cell Nrf2 protein expression. Compared to the that in the control group, WPC alone and combined treatment resulted in reduced expression of GCLC and GR proteins. However, treatment with rapamycin alone had no effect on the expression of these proteins.

### Rapamycin-induced nuclear accumulation of Nrf2 in MDA-MB-231 cells is reduced by WPC treatment

**Figure 5:**
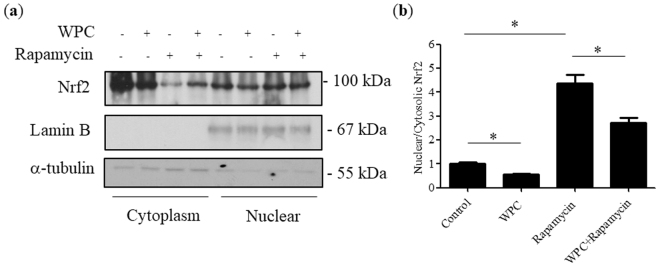
Effect of when protein concentrate (WPC), rapamycin, and their combination on Nrf2 translocation in MDA-MB-231 cells. (**a**) Representative western blots showing the effect of 48 hours of exposure to 10 mg/mL WPC, 1 μM rapamycin, and their combination on cytosolic and nuclear Nrf2 protein levels. α-tubulin and lamin B were used as loading controls for cytosolic and nuclear fractions, respectively. (**b**) Bar graphs showing relative nuclear/cytosolic Nrf2 ratios. Data represent the means ± SD from three experiments. *P < 0.05.

Since there were no significant differences in whole-cell Nrf2 protein expression among groups, we next evaluated the cellular translocation of Nrf2. As indicated in Fig. 5a and b, rapamycin treatment induced Nrf2 nuclear accumulation; however, WPC administration decreased the presence of Nrf2 in the nucleus, and inhibited the effect of rapamycin on Nrf2 translocation.

### WPC activates the GSK3/mTOR pathway

**Figure 6:**
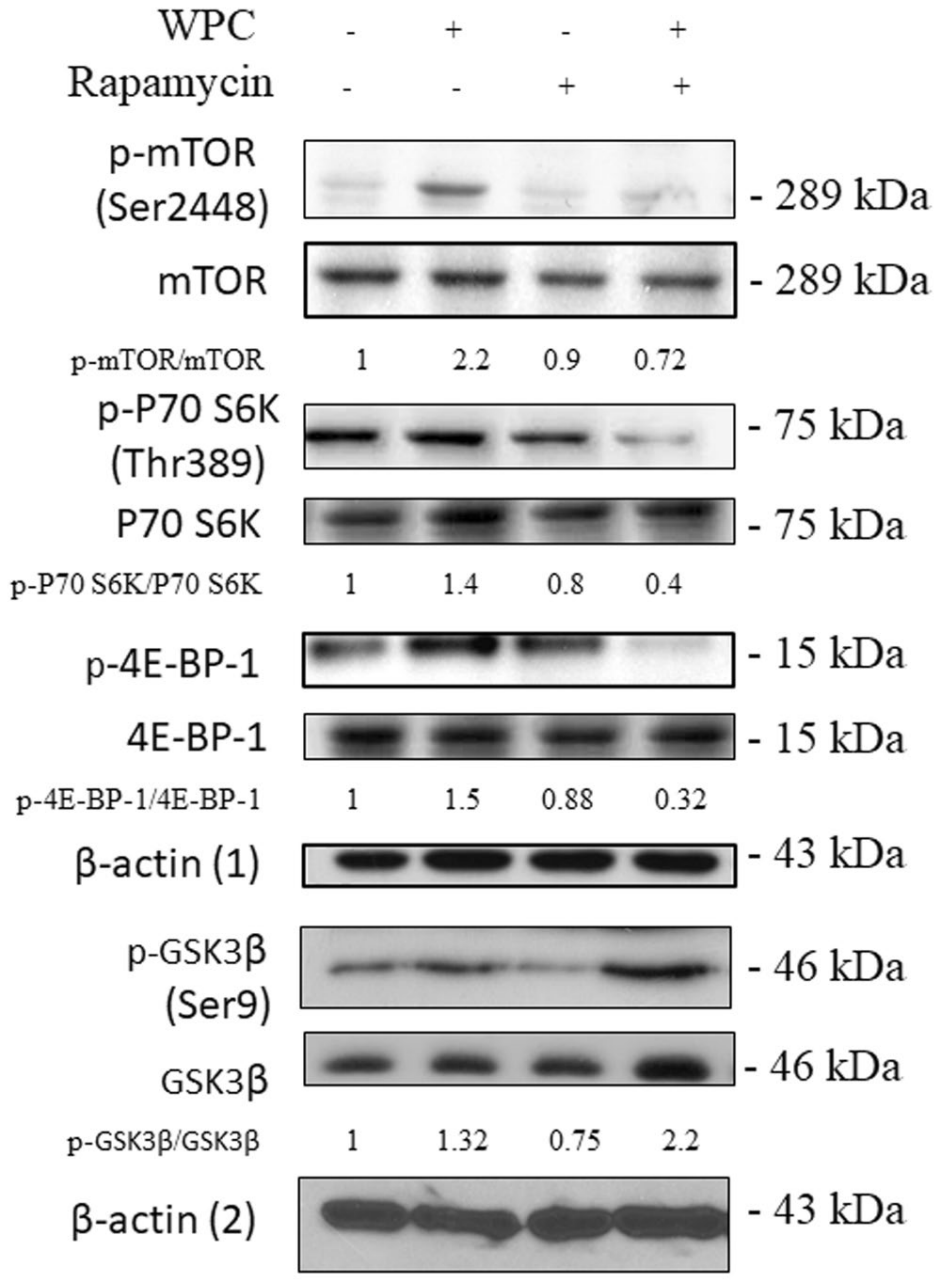
Effect of whey protein concentrate (WPC), rapamycin, and their combination on the mTOR/GSK3β signaling pathway in MDA-MB-231 cells. Representative western blots showing levels of p-mTOR (Ser 2448), mTOR, p-p70S6K (Thr 389), p70S6K, p-4E-BP-1, 4E-BP-1, p-GSK3β, and GSK3β in cells treated with 10 mg/mL WPC, 1 μM rapamycin, or both for 48 hours. β-actin (1) was used as a loading control of p-mTOR (Ser 2448), mTOR, p-p70S6K (Thr 389), p70S6K, p-4E-BP-1, and 4E-BP-1. β-actin (2) was used as a loading control of p-GSK3β, and GSK3β Data represent the means ± SD from three experiments.

We next assessed the combined effects of WPC and rapamycin on mTORC1 in MDA-MB-231 cells, since this complex is the direct target of rapamycin and is activated by nutritional factors. The level of phosphorylated (p)-mTOR (Ser2448) is a marker of mTORC1 activation. As shown in Fig. 6, the p-mTOR/mTOR ratio was approximately 2.2-fold higher in the WPC-only group compared to that in the control group. In contrast, compared to that in the control, the p-mTOR/mTOR ratio was found to be reduced with combined treatment. In addition, the p-p70S6K/p70S6K and the p-4E-BP1/4E-BP1 ratios were 1.4-fold and 1.5-fold higher, respectively, in the WPC-only group than the control group, whereas this effect was negated by the administration of rapamycin together with WPC. Strikingly, relative to that in the control, WPC upregulated p-GSK3β/GSK3β, both alone (1.32-fold) and with combined treatment (2.2-fold).

### WPC reduces Nrf2 nuclear accumulation and induces mTORC1 activation via GSK3β activity

**Figure 7:**
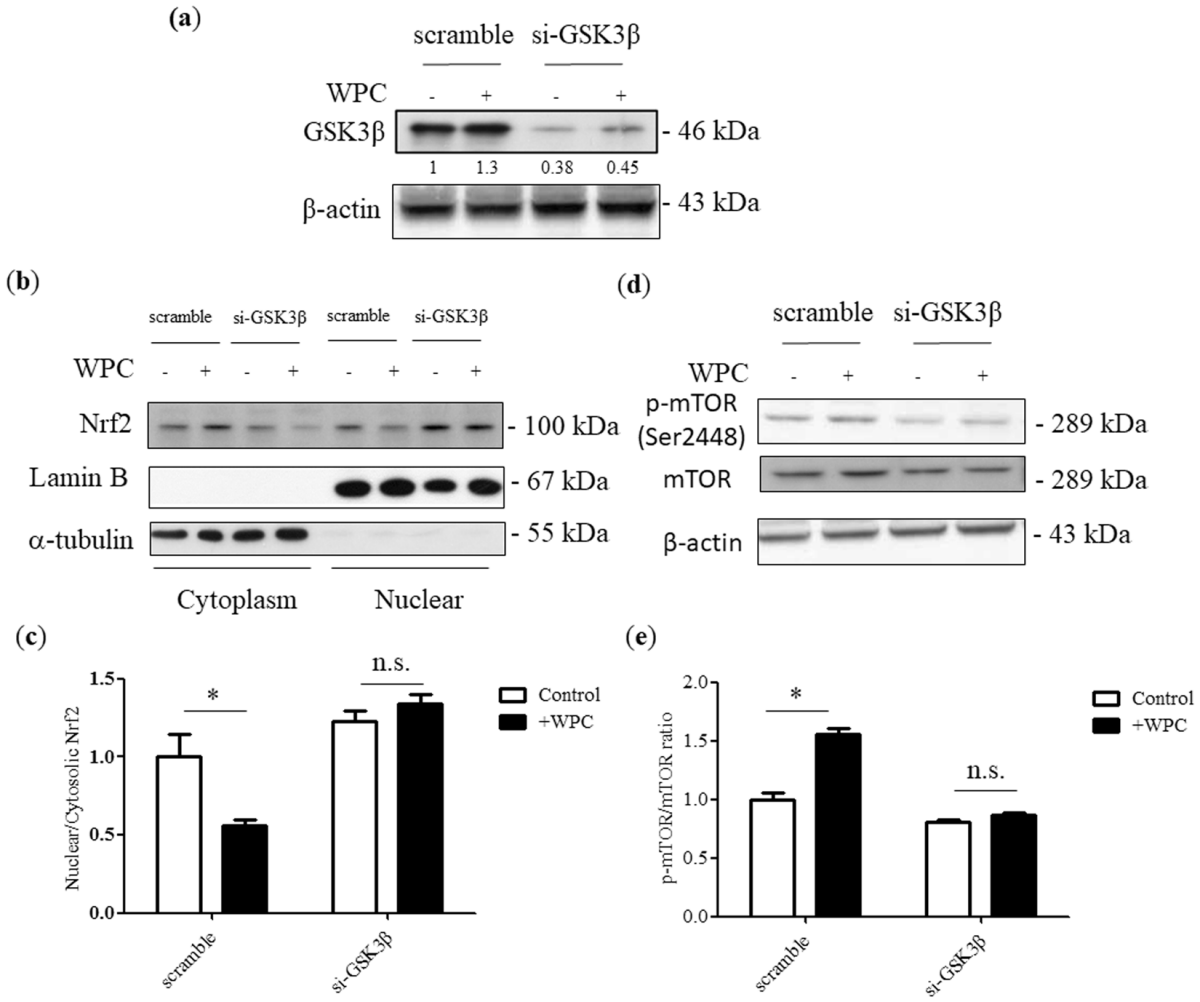
Whey protein concentrate (WPC)-induced reduction of Nrf2 nuclear accumulation and mTORC1 activation are disrupted by GSK3β siRNA. MDA-MB-231 cells were transfected with non-specific siRNA or GSK3β-targeting siRNA and treated for 48 hours with 10 mg/mL WPC. (**a**) Representative western blots showing GSK3β protein levels. β-actin was used as a loading control. (**b**) Representative western blots showing cytosolic and nuclear levels of Nrf2. Lamin B and α-tubulin were used as loading controls for nuclear and cytosolic fractions, respectively. (**c**) Bar graphs showing relative nuclear/cytosolic Nrf2 ratios. (**d**) Representative western blots demonstrating levels of p-mTOR (Ser2448) and mTOR. β-actin was used as a loading control. (**e**) Bar graphs displaying relative p-mTOR/mTOR ratios. Data represent the means ± SD from three experiments. *P < 0.05; n.s.: not significant.

To determine the mechanism underlying the WPC-mediated reduction in Nrf2 nuclear accumulation, we downregulated GSK3β protein expression using siRNA specific to the corresponding mRNA. Cells were transfected with non-specific (scrambled) siRNA or GSK3β siRNA, and were then treated with WPC. Protein expression was then detected by immunoblotting (Fig. 7a). GSK3β was inhibited, as expected, and this was found to result in increased nuclear Nrf2, compared to cytosolic levels (Fig. 7b,c). Moreover, cells treated with GSK3β siRNA exhibited a reduced p-mTOR/mTOR ratio in response to WPC treatment (Fig. 7d,e).

## Discussion

Although many studies have investigated molecular markers such as VEGF^21^, EGFR^22^, and mTOR^23^ as drug targets for TNBC, a greater understanding of the molecular basis of this disease is required for the development of effective treatments. In the present study, we demonstrated that WPC treatment renders MDA-MB-231 cells susceptible to rapamycin by (i) altering the cellular redox state and (ii) activating GSK3β/mTORC1 signaling.

Basal-like TNBC cells were reported to be highly sensitive to the mTOR inhibitor everolimus, whereas a subgroup of TNBC cells, comprising lines characterized as stem-like cancer cells, are less sensitive to this drug^24^. Given that the cellular redox state affects the regulation of signal transduction pathways and the development of drug resistance, this could be used to dictate therapeutic strategies for TNBC^25^. Many studies have shown that redox mechanisms regulate mTORC1 activity^26^. For example, Sarbassov *et al*. demonstrated that cysteine oxidants such as phenylarsine oxide and diamide activate mTORC1 *in vivo* and *in vitro* ^27^.

In the current work, we first observed that GSH and ROS levels were elevated in MDA-MB-231 cells compared to those in non-tumorigenic MCF-10A cells. Our previous results indicated an imbalance in GSH redox status in breast cancer patients. The high GSH levels observed in breast cancer tissues might be associated with enhanced cell proliferation and resistance to oxidative stress, representing a selective growth advantage for tumor cells over their normal counterparts^28^. In addition, Panis *et al*. reported that compared to adjacent normal breast tissue, tumors are sites of elevated oxidative and nitrosative stress, with concomitant augmented antioxidant capacity^29^.

WPC constitutes a rich source of bioavailable cysteine that can be used for GSH synthesis, and contains all nine essential amino acids. It is used to promote muscle protein synthesis and as a nutritional supplement for chemotherapy patients^30^. The PTEN-positive cell line MDA-MB-231 has been reported to be resistant to mTOR inhibition; however, we observed here that WPC treatment rendered these cells sensitive to rapamycin. Furthermore, WPC selectively altered GSH levels, affecting those in MDA-MB-231 cells, but not those in non-tumorigenic MCF-10A cells. This might be explained by the fact that GSH synthesis is regulated by negative feedback. Since GSH levels are much higher in tumor cells than in normal cells, it is easier to reach the threshold at which GSH synthesis is inhibited by negative feedback in the former than in the latter^31,32^. In addition, we found that treatment with 1 μM rapamycin, with or without WPC, induces ROS production in MDA-MB-231 cells. The relationship between rapamycin and ROS remains disputed, with some studies suggesting that rapamycin reduces cellular ROS levels^33^ and others claiming the opposite^34^. Such discrepancies might be explained by the important effect of rapamycin concentration^35,36^. In addition, there were no obvious changes in the viability of cells with WPC treatment. It is notable that in the present work, WPC selectively affected GSH levels but did not change cellular ROS content and viability, implying that a synergistic antitumor effect might be achieved by decreasing GSH levels in tandem with WPC treatment^37^.

Nrf2 regulates the inducible antioxidant program in response to cellular stress^38^. Here, we revealed that rapamycin treatment induces the nuclear accumulation of Nrf2, consistent with the observations of other researchers. It has also been reported that TORC1 inhibition by rapamycin increases transcription of the *Nrf2* gene^39,40^. Notably, combining rapamycin with WPC negated the promotion of Nrf2 nuclear accumulation by the rapamycin treatment in the present study. Moreover, we observed reduced expression of GCLC and GR proteins when cells were treated with WPC. Consistent with the observations of our previous *in vivo* study, we found that WPC supplementation reduced the nuclear accumulation of Nrf2 in tumor tissue^16^. Kerasioti *et al*. indicated that whey protein exerts its antioxidant activity through an Nrf2-dependent mechanism in endothelial cells; however, it was cell type dependent^18^.

It has been suggested that p-p70S6K^low^/p-4E-BP1^high^ cancer cells might be intrinsically resistant to mTORC1 inhibitors^41^. Of note, we found here that WPC treatment reversed this phenotype by enhancing phosphorylation of mTOR (Ser2448), p70S6K (Thr389), and 4E-BP1. Furthermore, we revealed that WPC upregulated GSK3β expression, consistent with a study by Stretton *et al*., in which GSK3β activity was shown to be required for the support of mTORC1 signaling in response to nutrient availability^4^. It has also been recognized that amino acid treatment promotes mTORC1 activation^42,43^. Our investigation suggests a combined therapy that might be beneficial for the subgroup of TNBC patients with tumors resistant to mTOR inhibitors.

Finally, in the present study, we inhibited GSK3β activity using siRNA, the results of which suggested that GSK3β is required for the reduction in Nrf2 nuclear accumulation mediated by WPC treatment. This is consistent with a report that activation and direct inhibition of GSK3β leads to nuclear exclusion and accumulation of Nrf2, respectively^44^. In addition, GSK3β was found to be associated with WPC-induced mTORC1 activation. Taken together, our results indicate that WPC leads to the depletion of GSH by reducing Nrf2 nuclear accumulation and activating GSK3β/mTOR signaling, rendering MDA-MB-231 cells susceptible to rapamycin.

**Figure 8:**
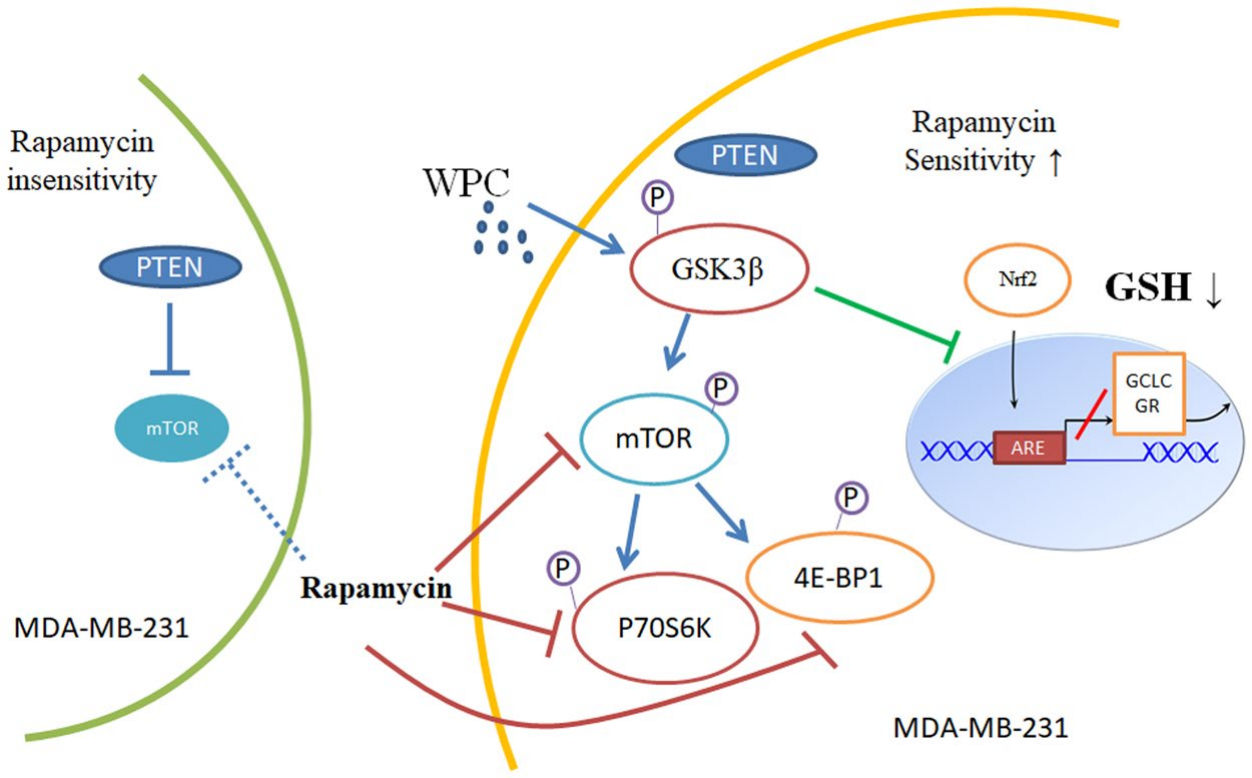
A working model of the mechanism through which whey protein concentrate (WPC) renders MDA-MB-231 cells sensitive to rapamycin. MDA-MB-231 cells express wild-type PTEN and are insensitive to rapamycin. WPC treatment activates mTOR/GSK3β signaling, involving increased p-p70S6K and p-4E-BP1 levels. In addition, activation of GSK3β inhibits translocation of Nrf2 from the cytoplasm to the nucleus, altering the cellular redox state by downregulating the glutamate-cysteine ligase catalytic subunit (GCLC) and glutathione reductase (GR), and finally reducing glutathione (GSH) levels. Taking together, WPC treatment thus induces rapamycin sensitivity in MDA-MB-231 cells.

In conclusion, we explored the result of combining antioxidant and mTOR inhibitory effects, and clarified the underlying mechanism for the first time (Fig. 8). Our findings might serve as a reference for the development of such combination therapies for the treatment of breast cancer, and particularly TNBC.

## Materials and Methods

### Reagents and antibodies

The WPC (Immunocal, Immunotec Research Ltd., Quebec, Canada) contained about 90% whey protein isolate, < 1.5% lactose, < 0.5% fat, and <5.0% moisture, with a solubility index of 99% at pH 4.6. WPC was freshly prepared before each experiment. The rapamycin and dimethylsulfoxide (DMSO) were purchased from Sigma-Aldrich (St. Louis, MO, USA). DMSO used as a solvent for rapamycin and control cells, which was not beyond 0.1%. The antibodies used in this experiment included: GCLC (abcam, ab53179), GR (abcam, ab16801), Nrf2 (Cell Signaling Technology (CST), #12721), mTOR (CST, #2972), p-mTOR (Ser 2448) (CST, #2971), p70S6K (CST, #9202), p-p70S6K (Thr389) (CST, #9205), 4E-BP1 (CST, #9644), p-4E-BP1 (Thr 37/46) (CST, #2855), GSK3β (CST, #9332), p-GSK3β (Ser9) (CST, #9336), β-actin (Sigma, A5441), α-tubulin (Santa Cruz Biotechnology, sc-8035), and Lamin B (sc-6216).

### Cell culture

The TNBC cell lines, MDA-MB-231 and MDA-MB-468 were obtained from American Type Culture Collection (Manassas, VA, USA). The nonmalignant MCF-10A breast epithelial cells were a kind gift from Taipei Medical University Hospital. MDA-MB-231 and MDA-MB-468 cells were cultured in DMEM supplemented with 10% fetal bovine serum (FBS), and MCF-10A cells were maintained in DMEM/F-12 medium supplemented with 5% horse serum, 0.5 μg/mL hydrocortisone, 100 ng/ml cholera toxin, 10 μg/mL insulin, and 20 ng/ml recombinant human EGF. All cells were cultured in a humidified incubator containing 5% CO_2_ at 37 °C.

### Preparation of WPC

Preparation of WPC was the same as in our previous study^15^. We followed the instructions of Immunocal to design the concentrations of WPC and WPC was freshly prepared before each experiment. Briefly, 50 mg of WPC was dissolved in a fresh 5 mL serum-free medium and centrifuged at 12,000 × *g* for 10 minutes at room temperature. The supernatant was filtered with a 0.2 μm filter. Therefore, concentrations of 10 mg/mL of WPC were used in this experiment.

### Determination of GSH

The 100 μL cell suspension, as indicated above, in an amber microcentrifuge tube was mixed with 0.1% MPA in a 1:2 volumetric ratio, and then it was centrifuged at 12,000 × *g* for 10 minutes after being adequately mixed by a vortex mixer. The supernatant was assayed on a capillary electrophoresis analyzer (P/ACE MDQ, Beckman Coulter) after being filtered with a 0.2 μm syringe set. The analysis was performed at a constant temperature (28 °C) with 300 mM borate running buffer (pH 7.8) and a UV absorbance detector set to 200 nm.

### Flow cytometric analysis of ROS levels

The intracellular ROS levels were assessed by flow cytometry using DCFH-DA. To assess the ROS levels, the cells were resuspended in PBS at 1,000,000 cells per mL and incubated in the presence of DCFH-DA (10 μM) in the dark at 37 °C for 30 minutes. Then, the cells were washed and resuspended in PBS. Finally, submitted the sample into flow cytometric analysis using a FACScan flow cytometer (Coulter EPICS XL Flow Cytometer, Beckman Coulter). Analyses were performed on 10,000 cells per sample and fluorescence intensities were measured on a logarithmic scale of four decades of log of fluorescence. Each experiment was repeated at least three times.

### Cell viability assays

Cell viability was assessed by 3-(4,5-Dimethylthiazol-2-yl)-2,5-diphenyltetrazolium bromide (MTT) dye conversion at 570 nm following the manufacturer’s instructions. Briefly, cells were seeded 5,000 per well in a 96-well flat bottom plate and grown for 24 hours. Cells were then treated for 48 hours or 72 hours with indicated concentrations of rapamycin and WPC in the presence of 10% FBS. 20 μl of MTT (5 mg/mL in PBS) was then added to each well and incubated 2 hours at 37 °C. The formazan in viable cells were dissolved with 100 μl of dimethyl sulfoxide and determined by reading optical densities in a microplate reader (Dynatech Laboratories, Chantilly, VA, USA).

### Colonies formation assay

MDA-MB-231 cells were seeded in 6-well plate at a density of 1,500 cells per well duplicate. These cells were cultured with respective treatment including WPC (2 mg/mL), rapamycin (200 nM), and their combination. The medium was replaced every 2–3 days until day 14. Colonies were stained with 0.2% (w/v) crystal violet in buffered formalin for 10 minutes. The number of colonies was counted.

### Preparation of cytosolic and nuclear extracts

Cytosolic and nuclear extracts were isolated according to Schreiber *et al*.^45^ with some modify cations. After rinsing with cold PBS, cells were resuspended in cold lysis buffer A (20 mM HEPES pH 8.0, 1 mM EDTA, 1.5 mM MgCl_2_, 10 mM KCl, 1 mM DTT, 1 mM sodium orthovanadate, 1 mM NaF, 1 mM PMSF, 0.5 mg/mL benzamidine, 0.1 mg/ml leupeptin, and 1.2 mg /mL aprotinin). They were incubated on ice for 15 min, after which 7.5 μL of 10% NP-40 detergent was added. Afterwards the cells were intensively stirred in a vortex mixer for 10 s. The homogenate was centrifuged at 15,000 × *g* for 50 seconds, and the supernatant was used as the cytosolic extract. The nuclear pellet was resuspended in cold extraction buffer B (20 mM HEPES pH 8.0), 1 mM EDTA, 1.5 mM MgCl_2_, 10 mM KCl, 1 mM DTT, 1 mM sodium orthovanadate, 1 mM NaF, 1 mM PMSF, 0.5 mg/mL benzamidine, 0.1 mg/mL leupeptin, 1.2 mg/mL aprotinin, and 20% glycerol). All protein fractions were stored at −70 °C until used.

### Western blotting

Cells were seeded, cultured, and treated with WPC (10 mg/mL), rapamycin (1 μM), and their combination for 48 hours. The proteins were separated by SDS-PAGE and transferred to polyvinylidene difluoride (PVDF) membrane. The membranes were blocked with 5% milk for 1 hour and incubated with targeted primary antibodies overnight at 4 °C, and then incubated with HRP-conjugated secondary antibodies at room temperature for 1 hour. Finally, the signals were enhanced by ECL (Millipore, Temecula, CA, USA) and exposed to X-ray film for autoradiogram. The intensity of the bands was quantified by densitometry and the results were evaluated relative to the expression of the constitutively expressed protein β-actin.

### Small interfering RNA (siRNA)

GSK-3β was silenced using siRNA (Santa Cruz Biotechnology, sc-35527) and a nonspecific scrambled siRNA (sc-37007) used as the negative control. Cells at 80% confluency were transfected with 2 μg of siRNA/well in 6-well plates using lipofectamine 2000 (Invitrogen). After they had been transfected, the cells were incubated for 18 hours in DMEM supplemented with 10% FBS at 37 °C, then treated with WPC or rapamycin for additional 48 hours.

### Statistical analysis

Data were expressed as mean ± standard deviation (SD) from three experiments. P-values were determined by Student’s *t*-test or one-way analysis of variance (ANOVA), followed by the Bonferroni post-test. Statistics were calculated using GraphPad Prism 5.0 software. Values of *P* < 0.05 was considered statistically significant.

## Electronic supplementary material

Supplementary Information
