# Supplementary material for: Whey Protein Concentrate Renders MDA-MB-231 Cells Sensitive to Rapamycin by Altering Cellular Redox State and Activating GSK3β/mTOR Signaling
Source: Sci Rep. 2017 Nov 21;7:15976. doi: 10.1038/s41598-017-14159-5 (PMC5698404; doi:10.1038/s41598-017-14159-5)
Supplement: Supplementary file 1 — Supplementary Information [file 41598_2017_14159_MOESM1_ESM.doc]

**Supplementary Information**

**Whey Protein Concentrate Renders MDA-MB-231 Cells Sensitive to Rapamycin by Altering Cellular Redox State and Activating GSK3β/mTOR Signaling**

Shih-Hsuan Cheng, Yang-Ming Tseng , Szu-Hsien Wu , Shih-Meng Tsai , Li-Yu

Tsai


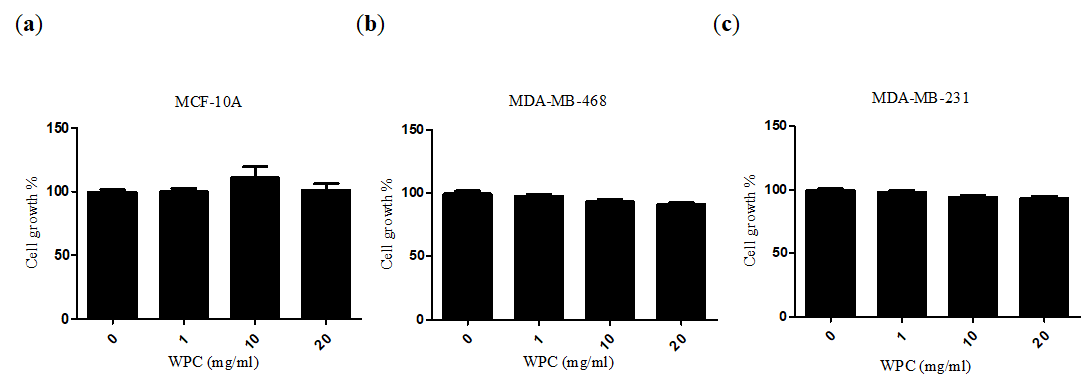


**Supplementary Fig. S1.**

**Effect of Whey protein concentrate** (**WPC) on cellular viability of MCF-10A, MDA-MB-468, and MDA-MB-231 cells. (a)** MCF-10A, **(b)** MDA-MB-468, and **(c)** MDA-MB-231 cells were treated with WPC at different concentrations for 48 hours, and cell viability was measured by standard MTT assay. Data represent the means ± SD from three experiments.
